# Dynamic metabolic modeling of a microaerobic yeast co-culture: predicting and optimizing ethanol production from glucose/xylose mixtures

**DOI:** 10.1186/1754-6834-6-44

**Published:** 2013-04-01

**Authors:** Timothy J Hanly, Michael A Henson

**Affiliations:** 1 Department of Chemical Engineering, University of Massachusetts, Goessmann Lab 159, 686 N. Pleasant St, Amherst, MA 01003-3110, USA

**Keywords:** Co-culture, Cellulosic ethanol, Fermentation, Mathematical modeling, *Saccharomyces cerevisiae*, *Scheffersomyces stipitis*

## Abstract

**Background:**

A key step in any process that converts lignocellulose to biofuels is the efficient fermentation of both hexose and pentose sugars. The co-culture of respiratory-deficient *Saccharomyces cerevisiae* and wild-type *Scheffersomyces stipitis* has been identified as a promising system for microaerobic ethanol production because *S. cerevisiae* only consumes glucose while *S. stipitis* efficiently converts xylose to ethanol.

**Results:**

To better predict how these two yeasts behave in batch co-culture and to optimize system performance, a dynamic flux balance model describing co-culture metabolism was developed from genome-scale metabolic reconstructions of the individual organisms. First a dynamic model was developed for each organism by estimating substrate uptake kinetic parameters from batch pure culture data and evaluating model extensibility to different microaerobic growth conditions. The co-culture model was constructed by combining the two individual models assuming a cellular objective of total growth rate maximization. To obtain accurate predictions of batch co-culture data collected at different microaerobic conditions, the *S. cerevisiae* maximum glucose uptake rate was reduced from its pure culture value to account for more efficient *S. stipitis* glucose uptake in co-culture. The dynamic co-culture model was used to predict the inoculum concentration and aeration level that maximized batch ethanol productivity. The model predictions were validated with batch co-culture experiments performed at the optimal conditions. Furthermore, the dynamic model was used to predict how engineered improvements to the *S. stipitis* xylose transport system could improve co-culture ethanol production.

**Conclusions:**

These results demonstrate the utility of the dynamic co-culture metabolic model for guiding process and metabolic engineering efforts aimed at increasing microaerobic ethanol production from glucose/xylose mixtures.

## Background

An essential component of the quest for energy independence is to develop renewable sources of energy via the conversion of plant biomass to liquid transportation fuels. Lignocellulosic biomass is a heterogeneous collection of polymers that compose plant cell walls, namely celluloses, hemicelluloses, pectins, lignins and proteoglycans [1]. The production of liquid fuels from biomass currently occurs in four major steps: pretreatment to make the feedstock more amenable to enzymatic degradation, hydrolysis of the cellulose and hemicellulose fractions to release sugar monomers and oligomers, fermentation of the released hexose and pentose sugars to produce fuels, and recovery of the fuels from the reactor bulk using separation technologies such as distillation [2,3].

The fermentation of sugar mixtures that result from biomass hydrolysis is a significant bottleneck in the overall process. Few fermentative microbes that efficiently convert both hexose and pentose sugars to liquid fuels such as ethanol have been identified [4,5]. The majority of current research efforts are focused on engineering multiple metabolic functionalities, such as the introduction of exogenous pentose consumption pathways, into a single organism [6]. However, this approach often results in conversion inefficiencies due to bottlenecks in metabolic pathways and may place a heavy metabolic burden on the organism [7,8]. In recent years, research has increasingly focused on the use of defined microbial consortia for biotechnology applications [9]. Microbial communities perform the task of biomass degradation in nature, albeit at a rate much slower than required for an industrial process [10]. Mixed cultures allow for the selection of microbes that are best suited for performing one task of the overall conversion process [11] and moves the engineering focus from introducing new functionalities to improving existing metabolic pathways. Other benefits of mixed culture systems include tunability and increased resistance to environmental stress [12,13].

*Saccharomyces cerevisiae* is a robust, budding yeast that has been widely used for fermentation of refined corn starch to fuel ethanol [14]. Because this yeast is Crabtree-positive, excess sugar that would otherwise overload its limited respiratory capacity overflows into the fermentative pathway. Thus, the yeast produces significant titers of ethanol when grown aerobically in batch culture [15]. However, *S. cerevisiae* is unable to utilize pentose sugars, such as xylose and arabinose, that result from the hydrolysis of hemicellulose. The engineering of pentose metabolism into the *S. cerevisiae* genome has been achieved, but problems with co-factor imbalances and gene expression have hindered the efficiency of these mutants [16].

Another species of yeast, *Scheffersomyces stipitis* (formerly known as *Pichia stipitis*), can natively ferment xylose to ethanol but it retains a preference for glucose as the carbon source. The growth and metabolite profile of this yeast is highly sensitive to the oxygenation level. Unlike *S. cerevisiae*, *S. stipitis* is a Crabtree-negative yeast that only produces ethanol under oxygen-limited conditions [17]. *S. stipitis* is among the most efficient native fermenters of xylose when grown under microaerobic culture conditions. In certain aeration regimes, however, this yeast can reassimilate ethanol while often simultaneously consuming other growth substrates [18]. Unlike most fermentative microorganisms, wild-type *S. stipitis* is unable to grow anaerobically. Insertion of the URA1 gene from *S. cerevisiae* has been shown to enable *S. stipitis* to grow anaerobically on glucose [19]. However, there are no known gene insertions that allow for *S. stipitis* anaerobic growth on xylose [20,21]. Thus, aeration level is a critical operating variable that must be tightly regulated to maximize xylose conversion to ethanol by wild-type *S. stipitis*.

*S. cerevisiae* and *S. stipitis* have been co-cultured for the production of ethanol from glucose and xylose mixtures [22-24]. In these studies, a respiratory-deficient strain of *S. cerevisiae* was used so the dissolved oxygen concentration could be more easily controlled at a level that was favorable for ethanol production by *S. stipitis*. Because it lacks the respiratory capability of a wild-type strain, respiratory-deficient *S. cerevisiae* could not utilize non-fermentable carbon sources such as ethanol once glucose had been exhausted [25]. The inability of these strains to grow on the ethanol produced under certain microaerobic conditions resulted in high ethanol titers. In fact, co-culturing these two yeasts on a mixture of glucose and xylose has been shown to yield more ethanol than can be produced by either yeast alone [23].

Constraint-based analysis using genome-scale metabolic reconstructions is a widely used computational tool for predicting how fluxes through microbial metabolic pathways will respond to changes in the culture environment or gene deletions/insertions [26,27]. With the addition of substrate uptake kinetics and extracellular mass balances on growth-limiting substrates and metabolic byproducts, these steady-state models can be adapted to predict culture dynamics that are critical in batch and fed-batch fermentations [28-30]. Recently, constraints-based modeling has been applied to mixed-culture systems [31-33]. In addition to describing individual species metabolism, mixed-cultures models must account for possible interactions between the species as well as postulate a community objective that captures the combined metabolic behavior. While the assumption that each species attempts to maximize its own growth rate is most common, other community objectives that capture more complex behavior such as altruism can be employed [34].

In this study, we used dynamic flux balance analysis (DFBA) to drive the experimental optimization of ethanol production from a respiratory-deficient *S. cerevisiae* and wild-type *S. stipitis* co-culture growing microaerobically on a mixture of glucose and xylose. A dynamic model of *S. stipitis* metabolism was developed from a recently published genome-scale reconstruction [20] by estimating glucose and xylose uptake parameters from batch pure culture data. Because the respiratory-deficient *S. cerevisiae* mutant had non-specific genetic alterations, we considered several plausible modifications of the wild-type metabolic network [35] to develop a *S. cerevisiae* dynamic model consistent with batch pure culture data. Co-culture experiments demonstrated that *S. cerevisiae* competed less successfully for glucose than expected from combining the pure culture dynamic models under the community objective of total biomass maximization. A revised co-culture model with reduced *S. cerevisiae* glucose uptake was shown to provide accurate predictions of batch co-culture data over a range of microaerobic growth conditions. The experimentally validated model was used to optimize the batch ethanol productivity by adjusting the inoculum concentrations and the aeration level. Finally, the model was used to examine what modifications to the *S. stipitis* xylose transport system would yield the largest improvements in co-culture ethanol production.

## Results and discussion

### S. stipitis pure cultures

Our initial dynamic model of *S. stipitis* metabolism did not include a balance on dissolved oxygen in the culture media. We were unable to satisfactorily fit this model to measured biomass and ethanol concentration profiles through adjustment of the sugar uptake rate parameters (see below). Because the dissolved oxygen (DO) concentration was above the assumed microaerobic value during the initial portion of the batch, we added the DO balance (8) to account for the apparent gas–liquid mass transfer limitations. The mass transfer coefficient (*k*_*L*_*a*) was determined from the gas sparge rate using a linear correlation (see Materials and Methods). Starting with literatures values when available [36], the uptake rate parameters for glucose (9), xylose (10) and oxygen (11) were determined by minimizing the least-squares difference between the experimental and predicted concentration profiles. Parameter adjustments were made by trial-and-error using glucose, xylose, biomass, and ethanol concentration profile measurements collected from two microaerobic batch fermentations performed at air sparging rates of 25 (*k*_*L*_*a* = 5.5 h^-1^) and 50 cc/min (*k*_*L*_*a* = 10.1 h^-1^). The resulting uptake parameters are compiled in Table 1.

**Table 1:** Substrate uptake rate parameters for pure and co-culture dynamic flux balance models

**Parameter**	***S. cerevisiae *** **311**	***S. stipitis***
v_g,max_ (mmol/gdw/h)	21.5 (pure culture) 18.5 (co-culture)	6.5
v_z,max_ (mmol/gdw/h)	-	5.5
v_o,max_ (mmol/gdw/h)	2.5	11
K_g_ (g/L)	0.5	1
K_z_ (g/L)	-	0.25
K_o_ (mM)	.005	0.0125
K_ieg_/K_iez_ (g/L)	10/-	10/4.5
K_igz_ (g/L)	-	0.5

**Figure 1:**
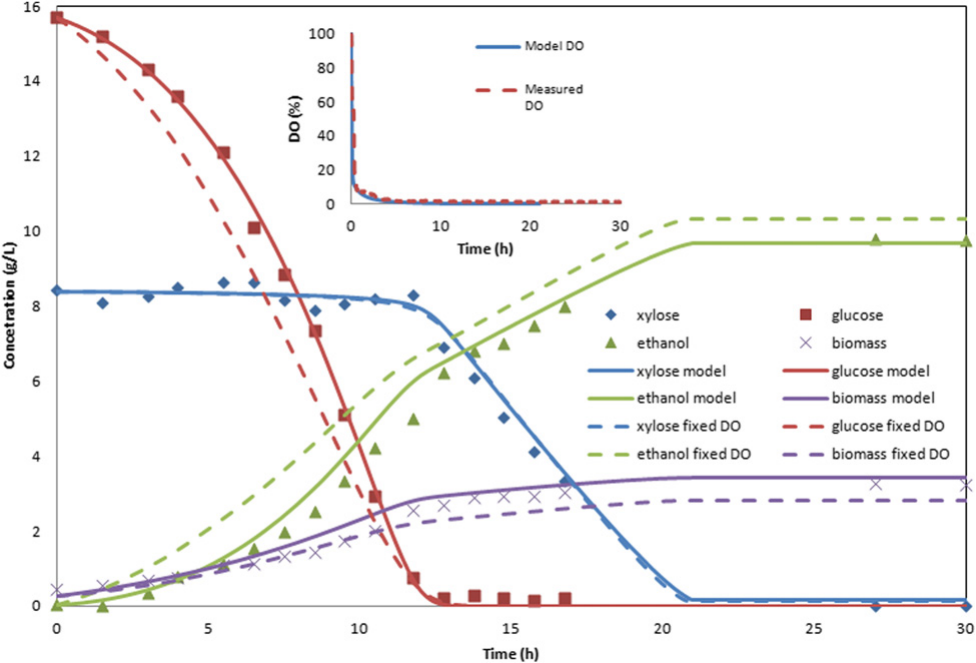
***S. stipitis *batch culture aerated at a *k***_***L***_***a *of 10.1 h**^**-1**^**.** Solid lines show dynamic model predictions when a balance on dissolved oxygen was included, while the dashed lines show dynamic model predictions at a fixed dissolved oxygen concentration of 0.00072 mM. Measured and predicted dissolved oxygen concentrations presented as percentage of the saturation value are shown in the inset.

**Figure 2:**
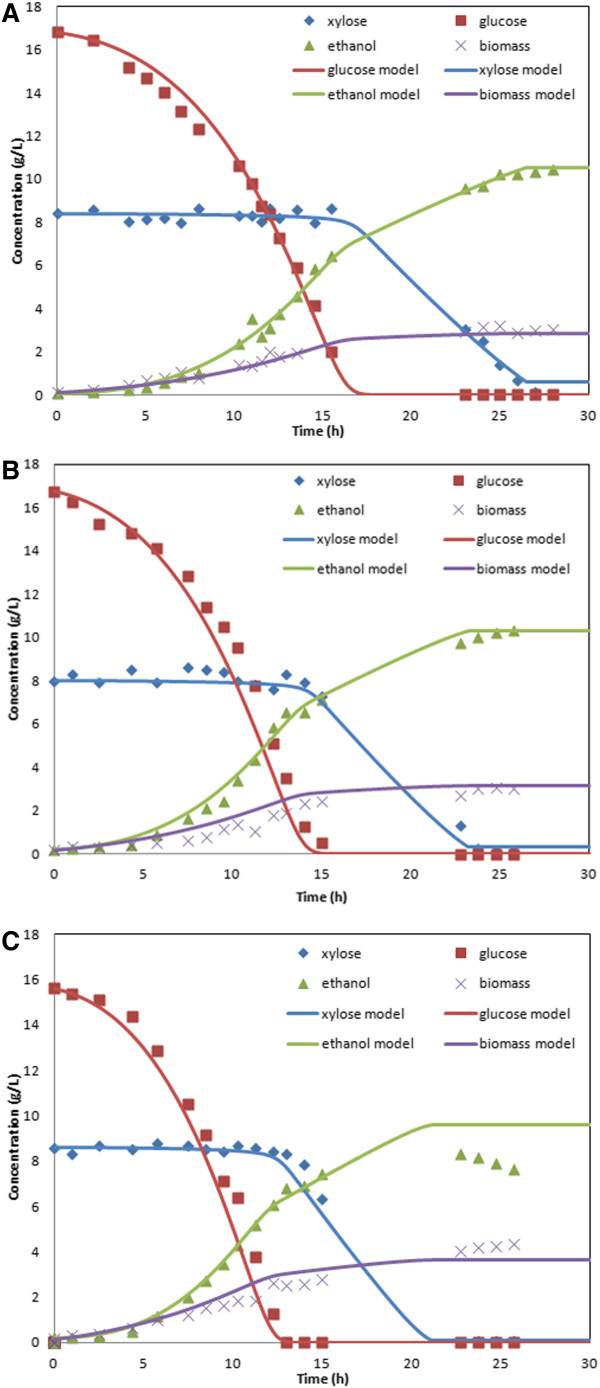
***S. stipitis *batch cultures aerated at a *k***_***L***_***a *of A) 5.5 h**^**-1 **^**B) 7.6 h**^**-1 **^**and C) 12.6 h**^**-1**^**.** Data points are experimental measurements, while solid lines are dynamic model predictions

The *S. stipitis* dynamic models with and without the DO balance (8) are compared in Figure 1 at an air sparging rate of 50 cc/min using initial conditions of 0.40 g/L biomass, 15.7 g/L glucose and 8.4 g/L xylose. A *k*_*L*_*a* value of 10.1 h^-1^ was used in the DO balance, while the model without the balance used a constant DO value of 0.0072 mM, the measured level at the end of the batch fermentation. The constant DO model could not reproduce the lag in ethanol production that resulted from the high level of dissolved oxygen present during the early stages of the fermentation. Additionally, the constant DO model produced lower biomass yields and higher ethanol yields than observed in experiment. Once the DO balance was added, the model could more accurately predict the time at which *S. stipitis* began microaerobic substrate consumption and ethanol production started to outpace biomass growth. The other *S. stipitis* pure culture experiment used for uptake parameter fitting of the variable DO model was performed with a *k*_*L*_*a* of 5.5 h^-1^ and initial conditions of 0.25 g/L biomass, 16.8 g/L glucose and 8.4 g/L xylose (Figure 2A). As before, the model produced very accurate predictions of the glucose, xylose, biomass and ethanol concentration profiles with the uptake parameter values in Table 1.

To examine extensibility of the *S. stipitis* dynamic model to different microaerobic conditions, batch experiments were performed at two additional *k*_*L*_*a* values and model predictions were generated with the same uptake parameter values listed in Table 1. Figure 2B shows a comparison of measured and predicted concentration profiles for a batch culture performed with a *k*_*L*_*a* of 7.6 h^-1^ and initial conditions of 0.25 g/L biomass, 16.8 g/L glucose and 8.0 g/L xylose. The simulation results were generally satisfactory, although the model predicted slightly faster consumption of both substrates and small overprediction of biomass production throughout most of the batch. A second validation experiment was performed with a larger *k*_*L*_*a* of 12.6 h^-1^ and initial conditions of 0.2 g/L biomass, 15.6 g/L glucose and 8.6 g/L xylose. As shown in Figure 2C, the model adequately captured the fermentation dynamics throughout the first 15 hours but it was unable to predict the subsequent reassimilation of ethanol that is evident in the data.

Although the *S. stipitis* dynamic metabolic model generated satisfactory predictions over a range of microaerobic conditions, the model failed to capture the ethanol and biomass concentration profiles at higher aeration levels once glucose had been exhausted. Ethanol can be simultaneously produced and reassimilated at these higher *k*_*L*_*a* values [18], resulting in overprediction of ethanol secretion and underprediction of biomass formation by our model. This phenomenon is likely due to the relative ease at which ethanol diffuses across the plasma membrane [37], while the uptake of xylose has been shown to be facilitated by oxygen [38]. Since xylose was more energetically favorable, the LP solver returned a flux distribution with simultaneous xylose and ethanol uptake only if there was excess oxygen available beyond that required for xylose metabolism. Even in absence of xylose, the *S. stipitis* metabolic reconstruction predicted that *k*_*L*_*a* values greater than 34 h^-1^ would be required just to meet the ATP maintenance demand for growth on ethanol. By contrast, we observed ethanol assimilation in our experiments for *k*_*L*_*a* values as low as 12.6 h^-1^. Without altering the objective function or the ATP maintenance coefficient of the iBB814 model, the observed disparity between model and experiment cannot be resolved. Fortunately, the highest ethanol yields and titers observed experimentally were obtained at aeration levels under which ethanol assimilation did not occur. As a result, we do not consider *k*_*L*_*a* values above ~10 h^-1^ in the remainder of the paper.

### S. cerevisiae 311 pure cultures

Because *S. cerevisiae* 311 was created by applying ethidium bromide to a wild-type strain, the genetic alterations that produced the respiratory-deficient mutant were non-specific. As a result, modifications to the wild-type iMM904 metabolic network necessary to describe *S. cerevisiae* 311 metabolism were unknown. We used the metabolic network model to identify putative gene knockouts consistent with the known alteration of mitochondrial DNA by ethidium bromide treatment [39] and to obtain predictions in agreement with our data. All the gene knockouts considered prevented growth on non-fermentable substrates and reduced oxygen demand. To determine the reactions to be deleted, model fluxes in the mitochondrial compartment were systematically set to zero until FBA simulations matched the *S. cerevisiae* 311 phenotype of lower biomass yields, higher ethanol yields and smaller oxygen demands than the wild-type strain. This process identified knockouts of genes encoding two enzymes: ubiquinol-6 cytochrome c reductase and mitochondrial cytochrome c oxidase. Modifications to these enzymes may well have occurred in *S. cerevisiae* 311, as other respiratory-deficient *S. cerevisiae* mutants have been created through the direct removal of the encoding genes [40].

**Figure 3:**
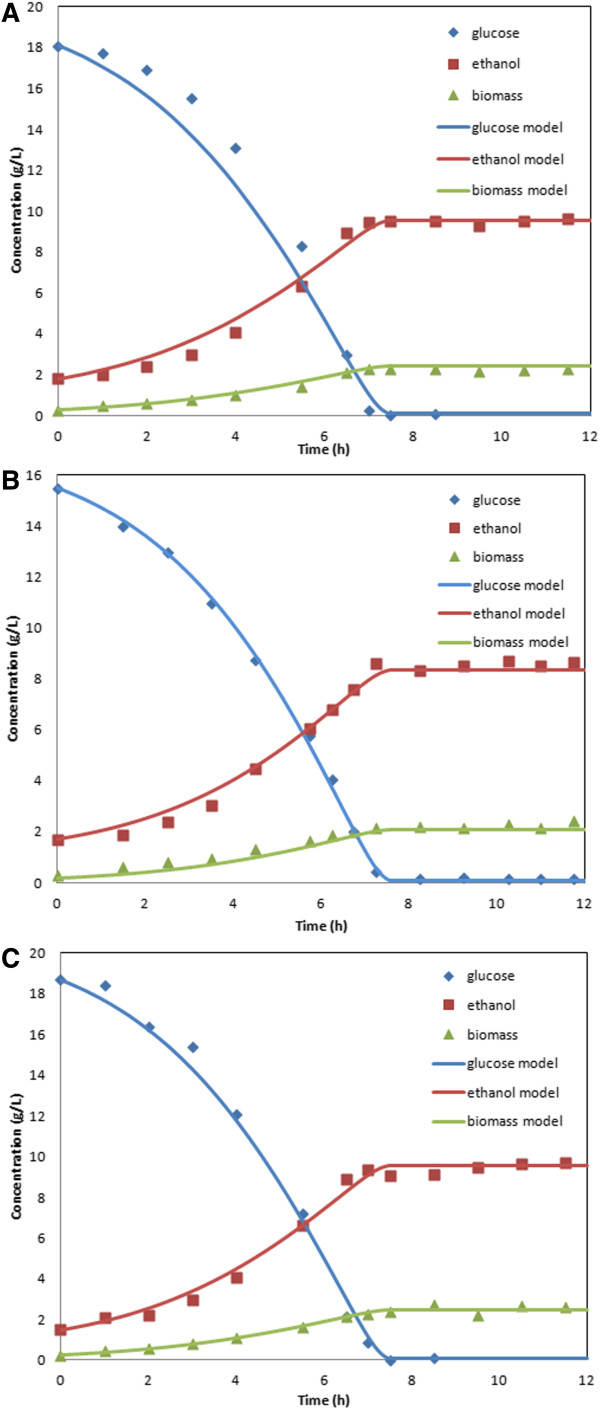
***S. cerevisiae *311 batch cultures grown A) anaerobically B) aerobically and C) at a *k***_***L***_***a *of 5.5 h**^**-1**^**.** Data points are experimental measurements, while solid lines are dynamic model predictions.

The *S. cerevisiae* 311 dynamic model was built on the modified iMM904 metabolic network with glucose uptake parameters estimated from anaerobic culture data and oxygen uptake parameters estimated from aerobic culture data with the glucose parameters fixed [41]. The resulting parameter values are listed in Table 1. Figure 3A shows a comparison of measured concentration profiles and model predictions for the anaerobic batch fermentation initiated with 0.28 g/L biomass, 18.0 g/L glucose and 1.8 g/L residual ethanol from the preculture shake flask. Figure 3B shows results for the aerobic fermentation initiated with 0.20 g/L biomass, 15.5 g/L glucose and 1.8 g/L ethanol. In each case, the dynamic model accurately predicted the entire ethanol profile and the glucose and biomass profiles in the second half of the batch. However, the model overpredicted glucose uptake and ethanol synthesis during the first half of the batch. While this discrepancy could have been attributable to the preculture cells used for inoculation being in stationary rather than exponential phase [42], we were unable to verify this hypothesis through additional experiments in which the preculture cells were harvested earlier.

To examine extensibility of the *S. cerevisiae* dynamic model to different aeration levels, a microaerobic batch experiment was performed at a *k*_*L*_*a* of 5.5 h^-1^ and model predictions were generated with the same uptake parameter values listed in Table 1. Figure 3C shows the results obtained for the fermentation initiated with 0.26 g/L biomass, 18.7 g/L glucose and 1.4 g/L residual ethanol. The model produced excellent agreement with data, and only small deviations in the biomass concentration during the initial portion of the batch and in the ethanol concentration following glucose exhaustion were observed.

### Co-culture modeling

A preliminary dynamic co-culture model was developed by direct combination of the two individual yeast models assuming no species interactions other than competition for glucose (see Materials and Methods). This approach resulted in faster glucose consumption and a higher final concentration of *S. cerevisiae* 311 than were observed in our microaerobic batch fermentations (not shown). We found that these discrepancies could be partially rectified by reducing the *S. cerevisiae* maximum glucose uptake rate from the pure culture value of 21.5 mmol/gdw/h to 18.5 mmol/gdw/h. Otherwise, the substrate uptake parameters remained fixed at the pure culture values listed in Table 1.

**Figure 4:**
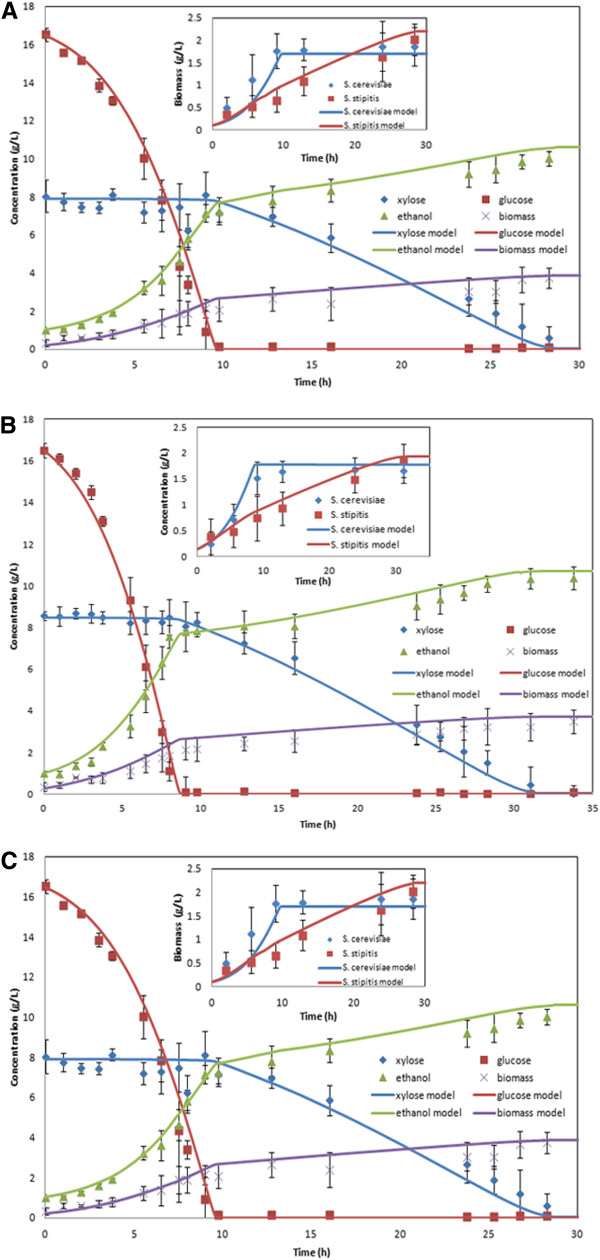
***S. cerevisiae*/*S. stipitis *batch co-cultures grown with an equal inoculum and aerated at A) 5.5 h**^**-1 **^**B) 9.6 h**^**-1 **^**and C) 7.6 h**^**-1**^**.** Individual cell concentrations are shown in the inset. Dynamic model predictions are indicated by solid lines. Three separate fermentations were performed at each aeration level to compute average values indicated by the symbols and coefficients of variation indicated by the error bars.

Figure 4A shows a comparison of the resulting model predictions and experimental data collected for three batch fermentations at a *k*_*L*_*a* of 5.5 h^-1^ with an equal inoculum of 0.15 g/L of each yeast species grown on 19.1 g/L glucose and 9.7 g/L xylose. Figure 4B shows corresponding results for a *k*_*L*_*a* of 10.1 h^-1^, 0.10 g/L of each yeast species, 16.5 g/L glucose and 7.9 g/L xylose. With the implemented change in the *S. cerevisiae* maximum glucose uptake rate, the dynamic co-culture model proved to be as accurate as the individual species models. The glucose consumption rate was slightly overpredicted during the initial portion of the batch at both aeration levels. Small discrepancies in the predicted biomass and ethanol concentrations were observed during the glucose consumption phase for *k*_*L*_*a* = 5.5 h^-1^, while small but longer lasting deviations were apparent in the biomass and xylose concentrations at *k*_*L*_*a* = 10.1 h^-1^.

To examine prediction accuracy of dynamic co-culture model at aeration levels not used for parameter adjustment, we performed three additional co-culture fermentations at a *k*_*L*_*a* of 7.6 h^-1^ with 0.145 g/L of each yeast as the inoculum and initial sugar concentrations of 16.5 g/L glucose and 8.5 g/L xylose. The model and data comparisons shown in Figure 4C are qualitatively similar to those obtained at the other two aeration levels. The results in Figure 4 collectively demonstrate that the co-culture model provided satisfactory predictions over a range of microaerobic conditions corresponding to *k*_*L*_*a* < ~10 h^-1^.

### Inoculum optimization

Following experimental validation, we utilized the dynamic co-culture model to computationally determine optimal bioreactor operating conditions for maximization of ethanol productivity. The productivity was defined as the final ethanol concentration divided by the batch time, which was chosen as the time at which the xylose concentration dropped below 0.5 g/L. Our simulations indicated that the aeration level had little effect on the ethanol titer but strongly affected the batch time due to the xylose consumption rate [38]. Our simulations also demonstrated that the relative amount of each yeast in the inoculum strongly affected the productivity through both the ethanol titer and the batch time. Therefore, ethanol productivity was optimized by adjusting the aeration level and inoculum concentrations. Rather than perform rigorous optimization [30], dynamic simulations were run with different combinations of the *k*_*L*_*a* value and the initial yeast concentrations, and the case that yielded the largest productivity was deemed the optimal solution. All simulations were performed with a mixture of 16.0 g/L glucose and 8.0 g/L xylose, while the total inoculum concentration was fixed at 1.0 g/L to allow direct comparison of results for different inocula.

**Figure 5:**
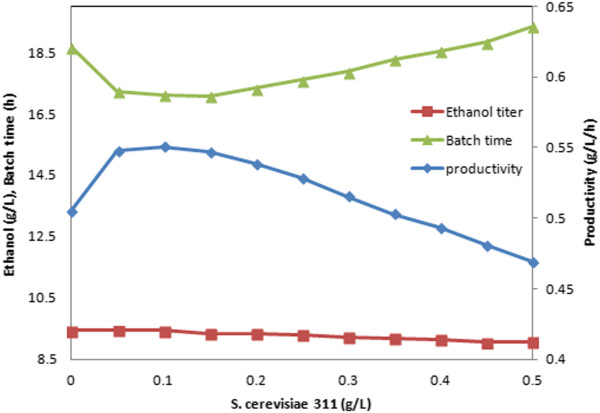
**Predicted effect of the inoculum concentration on batch co-culture performance measures at a *k***_***L***_***a *of 10.1 h**^**-1**^**.** The total inoculum concentration was fixed at 1 g/L.

The *k*_*L*_*a* value was constrained to be below 10.1 h^-1^ since larger values had the potential to result in undesirable ethanol reassimilation by *S. stipitis* that was not captured by the co-culture model. Regardless of the inoculum, we found that increasing *k*_*L*_*a* improved productivity due to a decrease in batch time that resulted from enhanced xylose consumption. Consequently, the optimal solution was achieved at *k*_*L*_*a* = 10.1 h^-1^, and the optimization problem was reduced to determining the inoculum concentrations. This result demonstrated the importance of developing improved metabolic reconstructions that more accurately predict the relationship between aeration level and the onset of ethanol reassimilation. Figure 5 shows the effect of the initial *S. cerevisiae* 311 concentration on the ethanol titer, batch time and ethanol productivity. The initial *S. stipitis* concentration is not shown since the total inoculum concentration was constrained to be 1.0 g/L. The ethanol titer increased with increasing *S. cerevisiae* concentration since this yeast converts glucose to ethanol at higher yields than *S. stipitis*. Conversely, the batch time decreased with increasing *S. cerevisiae* concentration because *S. stipitis* could better compete for glucose during the initial growth phase and the larger *S. stipitis* biomass concentration that resulted allowed for more rapid xylose consumption. These two competing effects produced a clear optimum in the ethanol productivity at 0.1 g/L *S. cerevisiae* and 0.9 g/L *S. stipitis*.

**Figure 6:**
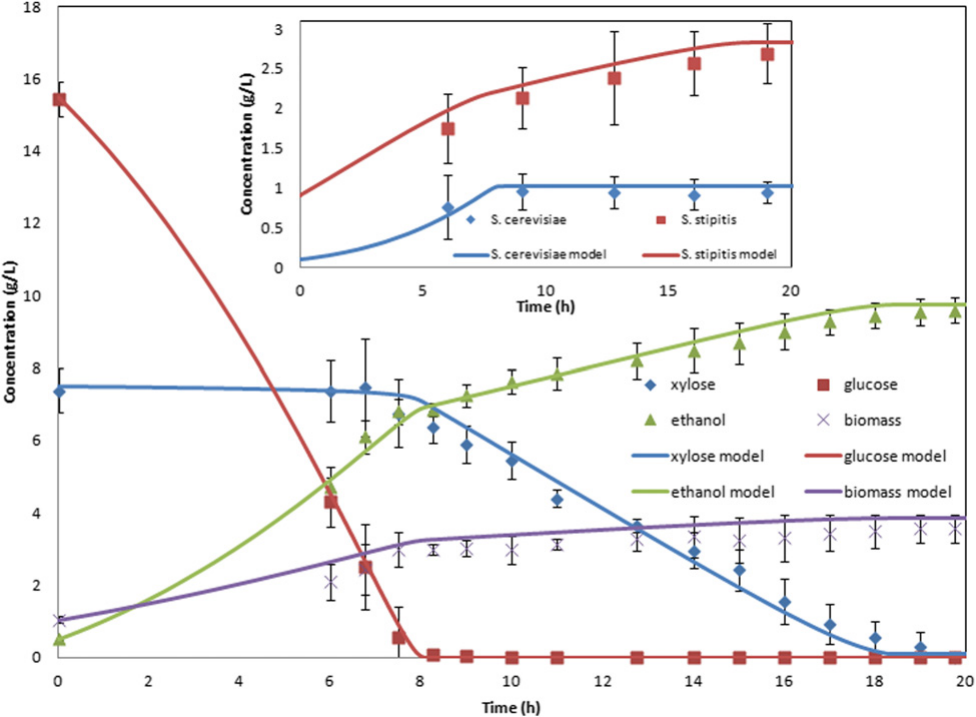
***S. cerevisiae*/*S. stipitis *batch co-cultures grown at the optimal conditions identified *in silico*.** The co-culture was inoculated with 0.1 g/L *S. cerevisiae* 311 and 0.9 g/L *S. stipitis* and aerated at a *k*_*L*_*a* of 10.1 h^-1^. Individual cell concentrations are shown in the inset. Dynamic model predictions are indicated by solid lines. Three separate fermentations were performed to compute average values indicated by the symbols and coefficients of variation indicated by the error bars.

To validate the model predictions, we performed three batch fermentations at the optimal conditions identified *in silico*. Figure 6 shows the averaged result of these experiments alongside the predicted growth and metabolite concentration curves. Although we were unable to obtain measurements during the first six hours, the optimized co-culture model produced excellent agreement with the measured concentration profiles after that time. The model predicted a final ethanol titer of 9.43 g/L and a productivity of 0.55 g/L/h, while the three fermentations were averaged to produce an ethanol titer of 9.07 g/L and a productivity of 0.521 g/L/h. According to our *in silico* analysis at *k*_*L*_*a* = 10.1 h^-1^, the optimized co-culture would outperform both a pure *S. stipitis* culture (9.43 g/L ethanol at a productivity of 0.505 g/L/h) and a co-culture with equal inoculum (9.07 g/L ethanol at a productivity of 0.469 g/L/h).

### In silico transporter engineering

Our simulations demonstrated that *S. stipitis* xylose metabolism was the rate limiting process that limited co-culture conversion efficiency. Moreover, xylose transport has been identified as the main bottleneck in pentose sugar metabolism with pure *S. stipitis* cultures [43]. Therefore, we used the dynamic co-culture model to predict the effects of modifying *S. stipitis* xylose transport parameters on ethanol productivity. To model engineering of the associated transport proteins, the nominal values listed in Table 1 for the maximum xylose uptake rate (*v*_*z,max*_), the xylose uptake saturation constant (*K*_*z*_), and the glucose inhibition constant for xylose uptake (*K*_*igz*_) were perturbed both upward and downward to determine their impact. The ethanol inhibition constant for xylose uptake (*K*_*iez*_) was excluded from detailed analysis because the ethanol concentrations achieved *in silico* were too small to cause significant xylose uptake inhibition. For the remaining three parameters, scaled sensitivity coefficients were calculated as:

(1) $$S=\frac{\bar{p}}{\bar{y}}\cdot \;\frac{\mathrm{\Delta y}}{\mathrm{\Delta p}}$$

where $\bar{p}$ is the nominal parameter value, Δ p is the parameter change, $\bar{y}$ is the optimal ethanol productivity obtained with the nominal parameter value, and Δ p is the predicted change in the ethanol productivity that results from the parameter change. All simulations were initialized with 16 g/L glucose and 8 g/L xylose at the optimum *k*_*L*_*a* = 10 h^-1^ and inoculum of 0.1 g/L *S. cerevisiae* 311 and 0.9 g/L *S. stipitis*.

**Figure 7:**
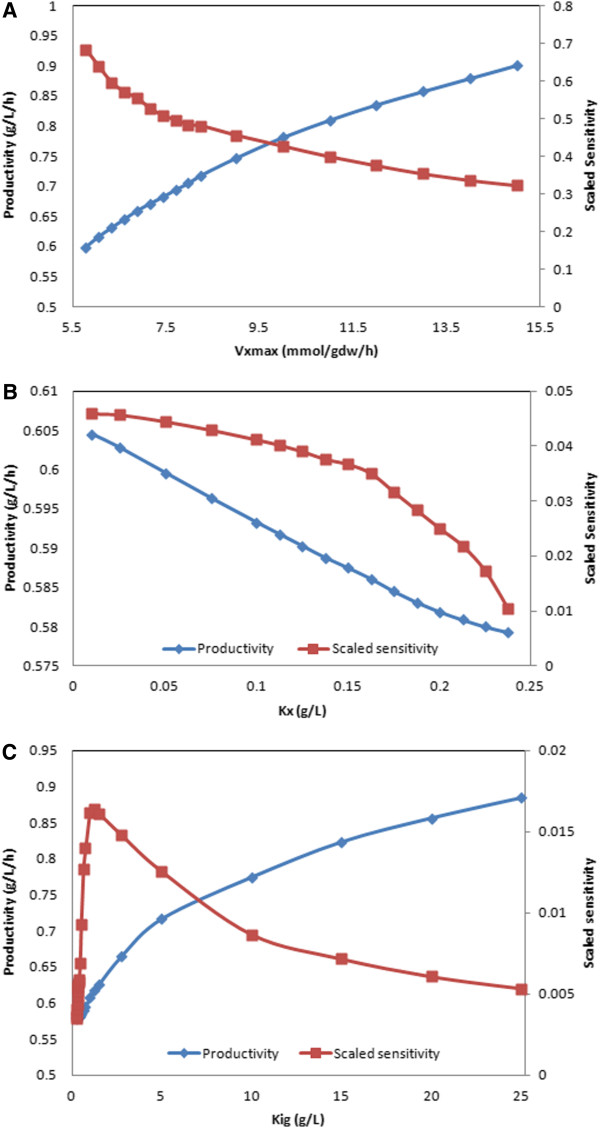
**Ethanol productivities and scaled sensitivity coefficients predicted by varying *S. stipitis *** **model parameters for the A) xylose maximum uptake rate (*v***_***z,max***_**) B) xylose uptake saturation constant (*K***_***z***_**) and C) glucose inhibition constant for xylose uptake.**

Figure 7 shows the ethanol productivities and scaled sensitivity coefficients obtained when *v*_*z,max*_ was changed upward and *K*_*z*_ and *K*_*igz*_ were changed downward. Increasing the maximum xylose uptake rate offered the largest improvement in ethanol productivity as indicated by the relatively large sensitivity coefficients, with a productivity enhancement of 14% achieved with a 25% *v*_*z,max*_ increase (Figure 7A). As indicated by the small sensitivity coefficients, more modest gains in ethanol productivity were predicted for decreases in the xylose uptake saturation constant (Figure 7B) and the glucose inhibition constant for xylose uptake (Figure 7C). However, complete elimination of *S. stipitis* diauxic growth by removal of the glucose inhibition term from the xylose uptake expression resulted in a large productivity enhancement of 85% (not shown). The simulated parameter changes could potentially be realized through protein engineering of the sugar transporters [44,45]. Because many *S. stipitis* transporters uptake both glucose and xylose [36], a complementary approach could be overexpression of specific transporters with more favorable xylose uptake characteristics and downregulation or deletion of less favorable transporters [46].

## Conclusions

Due to the ability of *Scheffersomyces stipitis* to efficiently convert pentose sugars to ethanol, co-cultures of *S. stipitis* and respiratory-deficient *Saccharomyces cerevisiae* are promising for renewable ethanol production from glucose/xylose mixtures [23,47]. Starting from genome-scale metabolic reconstructions of the individual yeasts, we developed a dynamic metabolic model of *S. cerevisiae*/*S. stipitis* co-cultures that was capable of predicting microaerobic metabolism in batch culture. As a first step towards co-culture modeling, dynamic models were developed for the individual yeasts using data from pure culture experiments. The Crabtree-negative yeast *S. stipitis* was shown to be very sensitive to the aeration level such that the incorporation of a dissolved oxygen balance was necessary to capture non-microaerobic oxygen levels during the initial batch phase and the subsequent switch from respiratory and to fermentative growth. The *S. stipitis* dynamic model was able to accurately reproduce measured glucose, xylose, biomass and ethanol concentration profiles over a range of microaerobic growth conditions characterized by oxygen-liquid mass transfer coefficient (*k*_*L*_*a*) values below ~10 h^-1^. However, the *S. stipitis* model was unable to capture ethanol reassimilation observed at higher oxygenation levels due to model energetics that required a *k*_*L*_*a* greater than 34 h^-1^ just to meet the ATP maintenance demand for growth on ethanol. Therefore, subsequent co-culture experiments and simulations were restricted to microaerobic conditions with *k*_*L*_*a* values less than ~10 h^-1^.

The procedure used to create the respiratory-deficient strain *S. cerevisiae* 311 introduced unknown genetic alterations that could not be directly implemented in the wild-type metabolic network. We used flux balance analysis to screen putative gene knockouts in the mitochondrial compartment that would reproduce the *S. cerevisiae* 311 phenotype of lower biomass yields, higher ethanol yields and smaller oxygen demands than the wild-type strain. The best agreement with *S. cerevisiae* 311 pure culture data was obtained for deletions of two genes that encode the ubiquinol-6 cytochrome c reductase and mitochondrial cytochrome c oxidase enzymes involved in the electron transport chain. With the fluxes through the two reactions catalyzed by these enzymes constrained to zero, the oxygen demand was greatly reduced because oxygen was not needed to serve as a final electron acceptor for ATP synthesis. These results were consistent with more directed genetic engineering efforts to create respiratory-deficient *S. cerevisiae* mutants through direct removal of these two genes [40].

A preliminary version of the dynamic co-culture model was developed by directly combining the dynamic models of the two yeast species under the assumption that each species attempted to maximize its individual growth rate and the only interspecies interaction was competition for glucose substrate. By comparison of model predictions to batch co-culture data collected at several microaerobic conditions, we found that the preliminary model overpredicted the glucose consumption and *S. cerevisiae* biomass formation rates. Much improved predictions were obtained by reducing the *S. cerevisiae* maximum glucose uptake rate from its pure culture value of 21.5 mmol/gdw/h to 18.5 mmol/gdw/h. This parameter change suggested the presence of unmodeled species interactions that resulted in *S. stipitis* having an antagonistic effect on *S. cerevisiae* growth. Crabtree-negative yeasts like *S. stipitis* have an established advantage when competing against a Crabtree-positive yeast such as *S. cerevisiae* for the same growth-limiting substrate [48]. This effect is further magnified when the Crabtree-positive species is a respiratory deficient mutant [49]. One possible cause for this effect was the competition for nutrients other than glucose. Growth under nitrogen limited conditions has been shown to slow the uptake of glucose and other hexoses in wild-type *S. cerevisiae*[50]. Respiratory-deficient *S. cerevisiae* could be at a disadvantage in competing for nitrogen sources such as ammonium when grown with respiratory competent *S. stipitis*. Because sugar uptake parameters reflect the action of numerous transport systems, each having distinct mechanisms and affinities, the decrease in this parameter may indicate differences in transporter expression between pure and co-cultures of the two microbes.

Following experimental validation, the dynamic co-culture model was used to compute the aeration level and initial cell concentrations that maximized batch ethanol productivity. Our *in silico* analysis suggested that the co-culture should be operated at the largest possible *k*_*L*_*a* value before the onset of ethanol reassimilation by *S. stipitis* because this aeration level maximized the rate of xylose conversion to ethanol. Because the *S. stipitis* metabolic network model did not accurately predict this transition point, the optimization was constrained by experimental data such that the optimal solution was achieved at *k*_*L*_*a* = 10.1 h^-1^. Optimization of the initial cell concentrations produced an inoculum with 90% *S. stipitis* and only 10% *S. cerevisiae* to form sufficient *S. stipitis* biomass following glucose exhaustion for efficient conversion of the remaining xylose. Experimental validation of the optimal solution showed that the co-culture model provided excellent agreement with measured concentration profiles. Compared to an unoptimized co-culture with equal initial cell concentrations, the optimized co-culture was shown experimentally to produce a 11% improvement in ethanol productivity. Additional *in silico* analysis indicated that the co-culture would yield higher ethanol productivities than a *S. stipitis* pure culture due to the higher glucose to ethanol conversion efficiency of *S. cerevisiae*.

The relatively slow conversion of xylose by *S. stipitis* is the main bottleneck that limits overall co-culture performance. The dynamic co-culture model was used to explore the potential impact of transporter engineering efforts aimed at enhancing xylose uptake by increasing the maximum xylose uptake rate, reducing xylose uptake saturation and reducing glucose inhibition of xylose uptake. Based on sensitivity analysis for moderate parameter changes, the maximum xylose uptake rate was predicted to yield the largest improvement in ethanol productivity. However, substantially larger improvements were predicted for complete elimination of glucose catabolite repression in *S. stipitis* because this modification eliminated the diauxic growth pattern and allowed xylose consumption to commence at the beginning of the batch. Taken together, our computational results suggest that an engineered *S. stipitis* strain which rapidly consumes xylose in the presence of glucose and does not reassimilate ethanol under microaerobic conditions would be an ideal candidate for enhancing co-culture performance. Future work should focus on the creation of such *S. stipitis* strains as well as the model-based characterization of co-culture performance for growth on actual biomass hydrolysates with inhibitory compounds.

## Methods

### Experimental

The wild-type *S. stipitis* strain NRRL Y-7124 (ATCC 58376) was used in this study. *S. cerevisiae* 311 (ATCC 42511), a mutant that was created by treating a wild-type strain with ethidium bromide [51], was chosen as the respiratory-deficient *S. cerevisiae* strain. Stocks of the two yeasts were stored at 4°C on YM agar slants.

All pure and mixed cultures were performed in a synthetic yeast minimal medium [52]. The composition per liter of water was 1.00 g MgSO_4_ ·7 H_2_O, 1.10 g/L KCl, 0.15 g CaCl_2_ · 2 H_2_O, 1.00 g (NH_4_)_2_HPO_4_, 8.75 g/L (NH_4_)_2_SO4, 60.3 mg myo-inositol, 30.0 mg Ca-panthothenate, 6.0 mg thiamine-HCl, 1.5 mg pyridoxine-HCl, 0.03 mg biotin, 10.6 mg MnSO_4_ · H_2_O, 9.0 mg ZnSO_4_ · 7 H_2_O, 5.0 mg FeSO_4_ · 7 H_2_O, and 2.4 mg CuSO_4_ · 2 H_2_O. Pre-cultures in media containing 20 g/L glucose and 20 g/L xylose for *S. cerevisiae* and *S. stipitis*, respectively, were grown at 30°C for 36 hours on a shake table set at 175 RPM. The inoculum concentration for each experiment was determined by calculating the volume of preculture required to obtain the target initial concentration of each cell type using the measured biomass concentration in the shake flask media.

All fermentations were performed in a HEL BioX array of 4 250 mL vessels situated in a shared block that provided both electric heat and independent magnetic agitation (HEL Group Ltd., Barnet, UK). Electrochemical probes monitored the dissolved oxygen and pH in each vessel, while individual thermocouples recorded the media temperatures. Bioreactor cultivations were performed at a constant temperature of 30°C and pH of 5, the optimal growth conditions for each yeast species [53]. The pH in each vessel was controlled by the automatic addition of 1 N sulfuric acid or 2 N NaOH. Glucose and xylose were autoclaved separately and added to the growth media in the amounts indicated for each experiment. Antifoam A was added to the reactors as necessary to prevent foaming.

Aeration of culture media was found to be a crucial operating variable. The agitation speed was held constant at 500 RPM for both pure and mixed culture fermentations. The gas flow rate into each reactor was altered according to the aeration level required for each experiment. A linear relationship between the gas sparge rate and the gas–liquid oxygen mass transfer coefficient (*k*_*L*_*a*) was determined using the static gassing out method [54]. Purely aerobic cultures were aerated with pure oxygen, while microaerobic fermentations were aerated with house air passed through a HEPA-VENT filter (Whatman Ltd., Kent, UK).

Total cell weight was measured using a correlation between OD595 measured on a WPA UV1101 Biotech Photometer (Biochrom Ltd.*,* Cambridge, UK) and dry cell weight. Cell counts of *S. cerevisiae* and *S. stipitis* in co-culture were performed on a hemacytometer in triplicate and averaged. A typical cell count considered approximately 50 *S. cerevisiae* cells and 200 *S. stipitis* cells. Conversion factors between dry cell weight and number of cells were found by drying pure culture samples of each yeast after cell counts had been performed. These factors were found to be 0.006943 gdw/L for *S. cerevisiae* and 0.001944 gdw/L for *S. stipitis*. Ethanol, glucose and xylose concentrations were measured by YSI 2700 SELECT biochemistry analyzers (YSI Inc., Yellow Springs, OH) configured with the enzyme-bound membranes specified for each metabolite. Raw readings were interpreted by 2700 Xylose PC Software (YSI Inc., Yellow Springs, OH) to resolve cross-talk between the xylose and glucose specific membranes.

### Modeling

The most comprehensive *S. cerevisiae* metabolic reconstruction currently available, iMM904 [55], was used for pure and mixed culture simulations. The fully compartmentalized network was reconstructed from 904 genes and accounts for 1228 metabolites and 1412 reactions. *S. stipitis* metabolism was simulated with iBB814 [20], the first published genome-scale reconstruction for this organism. This model accounts for 814 genes, 971 metabolites and 1371 reactions that are compartmentalized in the cytoplasm, mitochondria, and extracellular space. Following the publication of iBB814, a slightly more detailed *S. stipitis* metabolic reconstruction was developed [21]. We do not anticipate that the use of this alternative reconstruction would significantly alter the results reported in this paper.

The *S. cerevisiae*/*S. stipitis* co-culture model was constructed by combining the iMM904 and iBB814 stoichiometric matrices into a single matrix [56]. Flux distributions for *S. cerevisiae* (*v*_*c*_) and *S. stipitis* (*v*_*s*_) were calculated by solving the following linear program based on the assumption that the two species attempted to maximize their individual growth rates:

(2) $$\begin{array}{c} \begin{array}{cc} \max_{\;v_{c}{,v}_{s}} & \mu =\mu_{c}+\mu_{s}=w_{c}^{T}v_{c}+w_{s}^{T}v_{s} \end{array}\\ \left[\begin{array}{cc} A_{c} & 0\\ 0 & A_{s} \end{array}\right]\;\left[\begin{array}{c} v_{c}\\ v_{s} \end{array}\right]=\left[\begin{array}{c} 0\\ 0 \end{array}\right]\\ \;\left[\begin{array}{c} v_{c,\min}\\ v_{s,\min} \end{array}\right]\leq \left[\begin{array}{c} v_{c}\\ v_{s} \end{array}\right]\leq \left[\begin{array}{c} v_{c,\max}\\ v_{s,\max} \end{array}\right] \end{array}$$

where the subscript *i* represents the species, *A*_*i*_ is the matrix of stoichiometric coefficients, *v*_*i*_ is the vector of reaction fluxes including exchange fluxes, *v*_*i*,min_ and *v*_*i*,max_ are vectors of lower and upper flux bounds, *μ*_*i*_ is the growth rate, and *w*_*c*_ and *w*_*s*_ are vectors of experimentally determined weights that represent the contribution of each flux to biomass formation in *S. cerevisiae*[55] and *S. stipitis*[20], respectively. Other than competing for the common substrate glucose, the two yeasts were assumed to grow independently without species interactions. Therefore the co-culture objective function *μ* was assumed to be the sum of the individual species growth rates, and the inclusion of multi-level objective functions [34] was deemed unnecessary. The co-culture model was also used to simulate pure cultures of *S. cerevisiae* and *S. stipitis* by constraining all fluxes of the unmodeled organism to zero.

The steady-state flux balance model (2) was extended to a dynamic model through the addition of the following extracellular mass balance equations:

(3) $$\frac{dX_{c}}{\mathrm{dt}}=\mu_{c}X_{c}$$

(4) $$\frac{dX_{s}}{\mathrm{dt}}=\mu_{s}X_{s}$$

(5) $$\frac{\mathrm{dG}}{\mathrm{dt}}=-v_{g,c}X_{c}-v_{g,s}X_{s}$$

(6) $$\frac{\mathrm{dZ}}{\mathrm{dt}}=-v_{z,s}X_{s}$$

(7) $$\frac{\mathrm{dE}}{\mathrm{dt}}=v_{e,c}X_{c}+v_{e,s}X_{s}$$

(8) $$\frac{\mathrm{dO}}{\mathrm{dt}}=-v_{o,c}X_{c}-v_{o,s}X_{s}+k_{L}a\left(O*-O\right)$$

where *X*_*c*_ and *X*_*s*_ are the biomass concentrations of *S. cerevisiae* and *S. stipitis*, respectively, *G*, *Z*, and *E* are the concentrations of glucose, xylose, and ethanol, respectively, *v*_*e*,c_ and *v*_*e*,s_ are ethanol exchange fluxes, *v*_*g,c*_ is the glucose uptake rate for *S. cerevisiae*, and *v*_*g,s*_ and *v*_*z,s*_ are the glucose and xylose uptake rates, respectively, for *S. stipitis*. An equation for the dissolved oxygen concentration (*O*) was necessary to accurately describe microaerobic growth of *S. stipitis* (see results). In this equation (8), *v*_*o,c*_ and *v*_*o,s*_ are oxygen exchange fluxes, *k*_*L*_*a* is the volumetric mass transfer coefficient of oxygen from sparged gas to the culture medium, and O* is the saturation concentration of oxygen. For all simulations, O* was taken to be 0.24 mM, the saturation concentration for water at 30°C and 1 atm.

The following substrate uptake expressions were used to calculate upper bounds on the actual sugar and oxygen uptake rates:

(9) $$v_{g}=v_{g,\max}\frac{G}{K_{g}+G}\frac{1}{1+\frac{E}{K_{\mathrm{ieg}}}}$$

(10) $$v_{z}=v_{z,\max}\frac{Z}{K_{z}+Z}\frac{1}{1+\frac{G}{K_{\mathrm{igz}}}}\frac{1}{1+\frac{E}{K_{\mathrm{iez}}}}$$

(11) $$v_{o}=v_{o,\max}\frac{O}{K_{o}+O}$$

where *v*_*g*,*max*_, *v*_*z*,*max*_ and *v*_*o*,*max*_ are the maximum uptake rates of each substrate, *K*_*g*_, *K*_*z*_ and *K*_*o*_ are corresponding saturation constants, *K*_*ieg*_ and *K*_*iez*_ are ethanol inhibition constants, and *K*_*igz*_ is a glucose inhibition constant. The glucose (9) and xylose (10) uptake rates were assumed to follow Michaelis-Menten kinetics with an additional inhibitory term that reflects growth rate suppression at high ethanol concentrations [57]. The glucose inhibition term added to the xylose uptake kinetics accounted for diauxic growth where *S. stipitis* favors glucose over xylose as the carbon source. The oxygen uptake rate was calculated from a Michaelis-Menten expression based on the dissolved oxygen content of the medium [15].

Pure and mixed culture dynamic flux balance models were solved using the Mosek optimization toolbox (Mosek ApS, Denmark) to resolve the linear program for intracellular metabolism within Matlab (Mathworks, Natick, MA) [30]. Because *S. cerevisiae* could not meet the non-growth associated ATP maintenance demand during the xylose-only consumption phase, the maintenance flux was constrained to zero after glucose depletion to prevent the LP solver from returning zero fluxes for the *S. stipitis* network. Due to time-scale differences between the sugar and oxygen consumption rates, the differential equation system (3)--(8) exhibited a high degree of stiffness. To reduce the time required to generate large numbers of DFBA simulations for parameter fitting and *in silico* culture optimization, Matlab stiff ODE solvers ode15s and ode23tb were used to obtain approximate solutions. An ODE solver with greater accuracy, ode23, was used to generate model predictions once parameters had been estimated or an optimum had been determined. A typical co-culture batch simulation that was solved in two minutes with ode15s required five hours with ode23.

## Abbreviations

μi: Growth rate (1/h); Ai: Stoichiometric Matrix; E: Ethanol concentration (g/L); G: Glucose concentration (g/L); Kg: Glucose uptake saturation constant (g/L); Kie: Ethanol inhibition constant (g/L); Kigz: Glucose inhibition constant (g/L); kLa: Oxygen mass transfer coefficient (h^-1^); Ko: Oxygen uptake saturation constant (mM); Kz: Xylose uptake saturation constant (g/L); O: Dissolved oxygen concentration (mM); O*: Dissolved oxygen saturation concentration (mM); vc: *S. cerevisiae* Flux distribution (mmol/gdw/h); ve,c: *S. cerevisiae* ethanol flux (mmol/gdw/h); ve,s: *S. stipitis* ethanol flux (mmol/gdw/h); vg,c: *S. cerevisiae* glucose uptake rate (mmol/gdw/h); vg,max: Maximum glucose uptake rate (mmol/gdw/h); vg,s: *S. stipitis* glucose uptake flux (mmol/gdw/h); vi,max: Vector of upper bound flux constraints (mmol/gdw/h); vi,min: Vector of lower bound flux constraints (mmol/gdw/h); vo,c: *S. cerevisiae* oxygen uptake rate (mmol/gdw/h); vo,max: Maximum oxygen uptake rate (mmol/gdw/h); vo,s: *S. stipitis* oxygen uptake rate (mmol/gdw/h); vs: *S. stipitis* Flux distribution (mmol/gdw/h); vz,max: Maximum xylose uptake rate (mmol/gdw/h); vz,s: *S. stipitis* xylose uptake flux (mmol/gdw/h); wc: *S. cerevisiae* biomass reaction; ws: *S. stipitis* biomass reaction; Xc: *S. cerevisiae* concentration (g/L); Xs: *S. stipitis* concentration (g/L); Z: Xylose concentration (g/L).

## Competing interests

The authors declare that they have no competing interests.

## Authors’ contributions

TH carried out the fermentation and modeling work and drafted the manuscript. MH conceived of the study, and participated in its design and coordination and helped to draft the manuscript. Both authors read and approved the final manuscript.
